# Novel role of cardiovascular MRI to contextualise tuberculous pericardial inflammation and oedema as predictors of constrictive pericarditis

**DOI:** 10.3389/fcvm.2024.1329767

**Published:** 2024-03-18

**Authors:** L. J. Giliomee, A. F. Doubell, P. S. Robbertse, T. J. John, P. G. Herbst

**Affiliations:** ^1^ Division of Cardiology, Department of Medicine, Faculty of Medicine and Health Sciences, Stellenbosch University and Tygerberg Hospital, Bellville, South Africa; ^2^ Heart Unit, Mediclinic Panorama, Cape Town, South Africa

**Keywords:** TB pericarditis, constrictive pericarditis, pericardial inflammation, pericardial oedema, CMR, pericardial effusion, risk stratification, diastolic cardiac dysfunction

## Abstract

Tuberculosis (TB) and human immunodeficiency virus/acquired immunodeficiency syndrome have reached epidemic proportions, particularly affecting vulnerable populations in low- and middle-income countries of sub-Saharan Africa. TB pericarditis is the commonest cardiac manifestation of TB and is the leading cause of constrictive pericarditis, a reversible (by surgical pericardiectomy) cause of diastolic heart failure in endemic areas. Unpacking the complex mechanisms underpinning constrictive haemodynamics in TB pericarditis has proven challenging, leaving various basic and clinical research questions unanswered. Subsequently, risk stratification strategies for constrictive outcomes have remained unsatisfactory. Unique pericardial tissue characteristics, as identified on cardiovascular magnetic resonance imaging, enable us to stage and quantify pericardial inflammation and may assist in identifying patients at higher risk of tissue remodelling and pericardial constriction, as well as predict the degree of disease reversibility, tailor medical therapy, and determine the ideal timing for surgical pericardiectomy.

## Introduction

The pericardial sac surrounds the heart in a unique double-layered manner, containing a small amount of fluid between these layers, and it serves to both stabilise the heart and provide a favourable environment to ensure minimal friction during each cardiac cycle (1). Tuberculosis (TB), the most common cause of pericardial constriction in endemic areas (2–5), can however disrupt this favourable environment, leading to a state of severe inflammation dominated by maladaptive tissue remodelling, fibrosis, and calcification (6–8). As a result, the heart becomes encased with impaired diastolic cardiac filling, characterising constrictive pericarditis (CP) (9, 10).

Chronic CP represents an irreversible state of haemodynamic compromise and is an indication for pericardiectomy, as untreated cases exhibit poor outcomes (9). While potentially curative, pericardiectomy carries high surgical risk, with significant peri- and post-operative morbidity and mortality (9–12). Early diagnosis and treatment, particularly when pericardial constriction is less advanced and associated with less fibro-calcification, lead to better surgical outcomes (13). On the other hand, premature intervention carries the risk of exposure to unnecessary surgery, as the dynamic inflammatory component seen in effusive-constrictive pericarditis (ECP) is typically reversible (14, 15). Therefore, optimal surgical timing is crucial to ensure favourable patient outcomes, but it remains challenging and often elusive in clinical practice.

This case-based review aims to illustrate the potential of new cardiovascular magnetic resonance (CMR) imaging data, which enables the staging and quantification of pericardial inflammation (16), in offering significant improvements in prognostication by identifying patients at higher risk for developing pericardial constriction. It may also guide clinical decision-making to optimise medical and surgical interventions and assist in determining the optimal timing for pericardiectomy.

## Case 1

A 36-year-old human immunodeficiency virus (HIV)-uninfected man presented with a 2-week history of dyspnoea, constitutional symptoms, and clinical findings suggestive of a pericardial effusion.

The diagnosis of a large circumferential pericardial effusion was confirmed via transthoracic echocardiogram (TTE), and a pericardiocentesis was performed, which confirmed a definitive diagnosis of rifampicin-sensitive TB pericarditis (17). Despite maximal drainage of the pericardial effusion (>1,000 ml), constrictive physiology (18) persisted, further classifying this case as ECP (19).

He was subsequently initiated on the local first-line anti-tuberculous chemotherapy regimen (20) without adjunctive anti-inflammatory therapy and was scheduled for review at 3-month intervals.

At the 3-month follow-up, he exhibited clinical features of predominantly right heart failure despite being compliant with his anti-tuberculous chemotherapy, and a diagnosis of constrictive pericarditis was confirmed via TTE (18, 19, 21).

**Figure 1:**
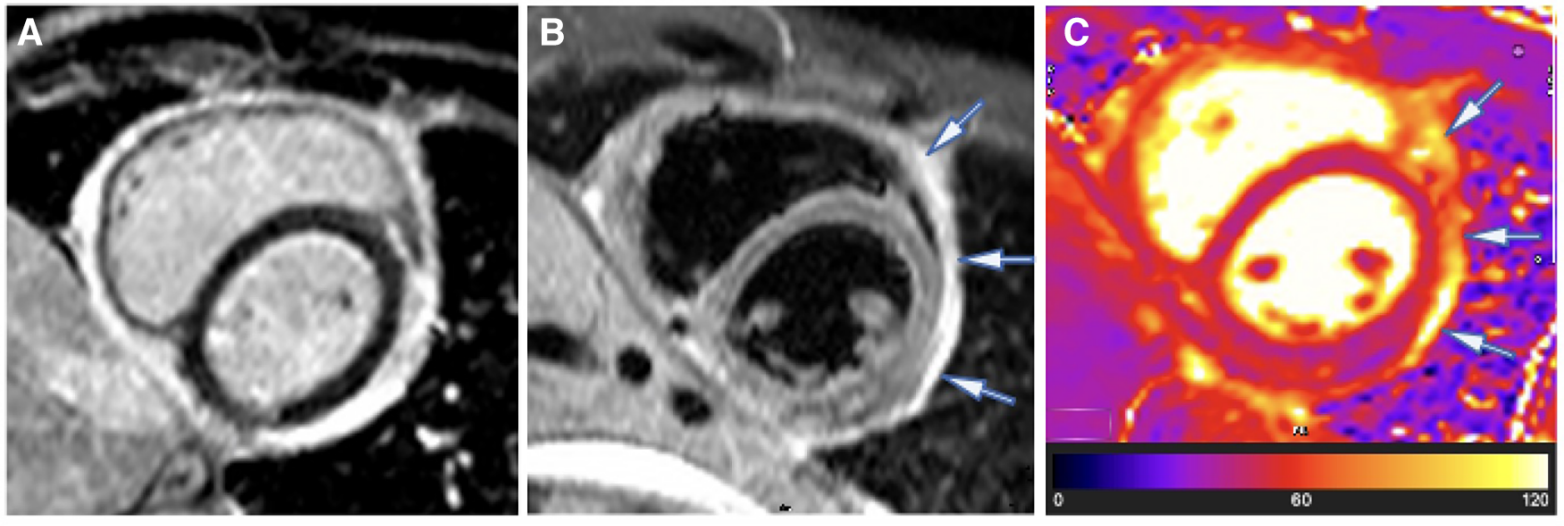
Case 1: 3-month CMR findings—sub-acute TB pericarditis with marked residual pericardial inflammation. (**A**) LGE image showing an intense circumferential pericardial signal indicative of marked circumferential pericardial inflammation. (**B**) T2-STIR and (**C**) T2 mapping images showing a high pericardial signal of segments overlying the LV lateral and anterior walls and anterior to the right ventricular (RV) free wall (indicated by arrows), with normal pericardial signal intensity of the other pericardial segments. Marked pericardial inflammation (**A**) demonstrates a risk of continued tissue remodelling and pericardial fibrosis; however, oedematous segments with acute pericarditis (arrows in **B**,**C**) represent reversible constrictive haemodynamics.

A CMR study was performed, which demonstrated severe residual pericardial inflammation, as evidenced by diffuse, circumferential hyperenhancement of the thickened pericardium observed on late gadolinium enhancement (LGE) imaging (see Figure 1A). Areas of residual pericardial oedema were also identified, as demonstrated by the focal areas of increased T2 short tau inversion recovery (STIR) and T2 mapping signal (see arrows in Figures 1B,C), without any re-accumulation of the pericardial effusion. In addition, CMR confirmed constrictive physiology on free-breathing real-time cine sequences (22–24).

Based on the presence of constrictive physiology 3 months after the initiation of anti-tuberculous chemotherapy and in accordance with expert consensus recommending optimal surgical timing to be 6–8 weeks after the initiation of anti-tuberculous chemotherapy, the patient was referred for surgical pericardiectomy (25).

However, the patient declined surgical intervention at this time, and the best medical therapy was continued (anti-tuberculous chemotherapy and diuretics) in addition to planning further clinical follow-up.

After 4 months of anti-tuberculous chemotherapy, the patient was found to be asymptomatic with complete resolution of symptoms and free from all signs of constrictive physiology on repeat TTE, despite the patient having stopped his diuretic therapy in the weeks preceding this follow-up.

**Figure 2:**
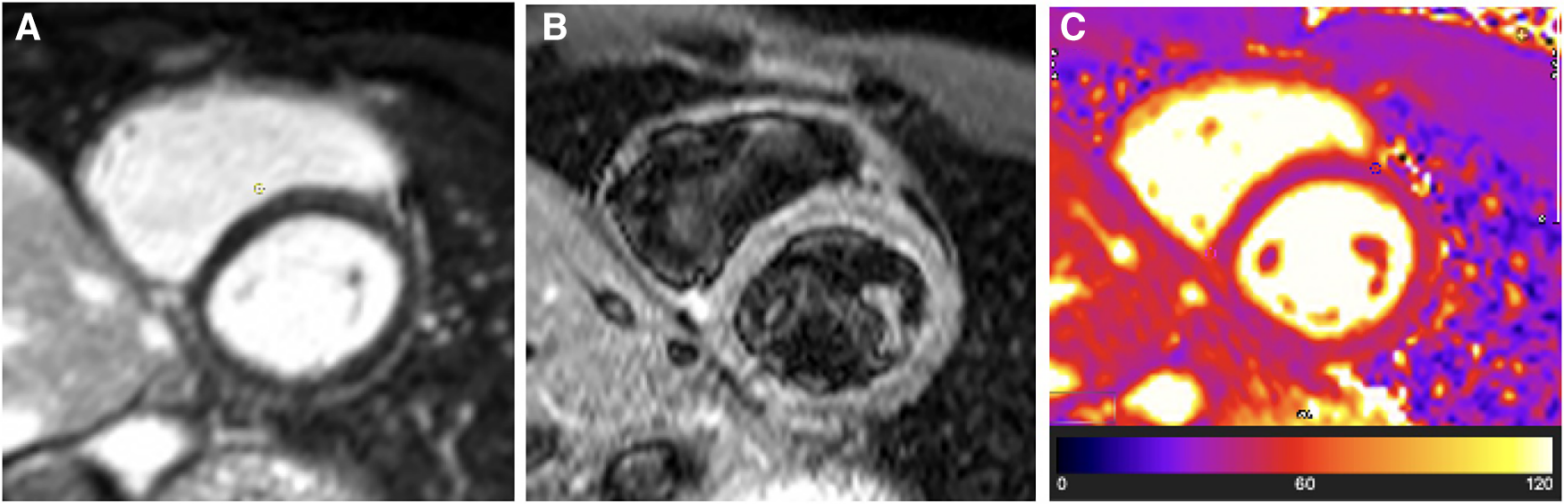
Case 1: 4-month CMR findings—burnt-out TB pericarditis. (**A**) LGE image showing normal pericardial signal intensity indicative of complete resolution of pericardial inflammation. (**B**) T2-STIR and (**C**) T2 mapping images showing normal pericardial signal intensities indicative of absent pericardial oedema. A burnt-out (resolved pericardial inflammation and oedema) CMR picture of TB pericarditis in the setting of non-constrictive haemodynamics is an indication that medical therapy can be stopped (or can be stopped as soon as the patient has completed the standard 6-month anti-tuberculous chemotherapy course) and that a pericardiectomy is not indicated.

Repeat CMR confirmed the complete resolution of pericardial oedema on T2-STIR imaging and T2 mapping (see Figures 2B,C), with minimal residual pericardial inflammatory signal seen on LGE sequences (see Figure 2A).

### Case 1: discussion

ECP is an umbrella term used to describe the presence of constrictive physiology after drainage of a large pericardial effusion (19). Increased pericardial stiffness in this setting results from a variable contribution of (1) acute pericardial inflammation and oedema and (2) established pericardial scarring, which cumulatively result in physiologically significant constrictive haemodynamics, persisting despite the drainage of pericardial fluid (16, 26, 27).

Constrictive haemodynamics is a term used to describe a specific form of physiologically significant haemodynamic compromise seen in pericardial constriction due to severe restriction of diastolic cardiac filling by the thickened and adhered pericardium (27). This is characterised by two important physiological principles: (1) dissociation of intrathoracic and intracardiac pressures, which drives respirophasic and reciprocal filling rates of the left and right ventricles, and (2) enhanced ventricular interaction due to respirophasic septal shift, which causes reciprocal ventricular filling of the left and right heart (27). These features are evident in pressure-volume haemodynamics and imaging modalities in patients with pericardial constriction (19). However, as seen in this case of ECP, the term “constrictive haemodynamics” does not specifically address the underlying pathophysiologic mechanism responsible for the increased pericardial stiffness leading to this impairment.

Echocardiography is widely regarded as the first-line investigation for the assessment of constrictive haemodynamics (25, 28, 29) and effectively demonstrates the principles of haemodynamic impairment through a combination of three echocardiographic measurements—also commonly known as the Mayo Clinic Criteria. (1) Respirophasic ventricular septal shift is used as a sensitive inclusion marker (87%) of constriction, whereafter a combination with either (2) medial e prime (e′) ≥9 cm/s (indicating normal intrinsic myocardial relaxation ability) or (3) hepatic vein early expiratory diastolic wave flow reversal ratio ≥0.79 is used to increase specificity to 91% (18).

Also, strain evaluation using 2D speckle tracking has yielded promising results (30). It illustrates that pericardial tethering (pericardial attachment to the epicardial cardiac surface pulling on the myocardium) causes decreased peak systolic strain in free cardiac walls (adjacent to the adherent pericardium), while septal peak systolic strain is maintained—the strain equivalent of annulus reversus seen on tissue Doppler. A left ventricular (LV) lateral wall to LV septal wall strain ratio of <0.96 was shown to diagnose constrictive haemodynamics with a sensitivity of 89% and a specificity of 96% (30).

Although effective in demonstrating the constrictive haemodynamics seen in ECP, its inability to differentiate between the underlying mechanisms responsible for constrictive haemodynamics remains a significant limitation. Therefore, whether the constriction is related to a transient, reversible process or whether it is chronic and irreversible cannot be judged by looking at the presence or absence of constrictive haemodynamics alone.

CMR is currently described as a second-line investigation for evaluating pericardial disease (25, 28). Apart from its ability to demonstrate constrictive haemodynamics with high sensitivity and specificity (22–24), CMR can delineate potential surgical dissection planes pre-operatively (seen as epicardial and pericardial separation by epicardial fat of variable thickness) and contribute to the risk assessment plan prior to pericardiectomy (28, 31). CMR’s unique ability to quantify and stage pericarditis, based on the presence of the distinct processes of pericardial inflammation and oedema, sets it apart from other imaging modalities and enables us to unravel the complex mechanisms underpinning constrictive haemodynamics (16).

Pericardial inflammation results in neovascularisation, fibroblast proliferation, and expansion of the pericardial extravascular space, causing the otherwise relatively avascular pericardium to accumulate and retain gadolinium-based contrast agents, leading to increased signal on LGE imaging (32, 33). Positron emission tomography (PET) data have shown this local pericardial inflammatory response to be especially intense in the case of TB pericarditis, where a pericardial inflammatory intensity [often quantified using the maximum standardised uptake value (SUVmax) on PET imaging] above a particular threshold was independently associated with a tuberculous aetiology (34).

Studies have also previously looked at the cytokine profile of this intense pericardial inflammatory response on a biochemical level, which demonstrated a pro-fibrotic inflammatory character dominated by high amounts of pro-inflammatory cytokines and low levels of anti-fibrotic N-acetyl-seryl-aspartyl-proline (Ac-SDKP) (35–37). Specifically, interleukin-1 beta (IL-1B), tumour necrosis factor-alpha (TNF-α), and transforming growth factor-beta (TGF-β) are detectable in high quantities within TB pericardial effusions, and these have been closely associated with fibrosis in the setting of chronic inflammatory diseases, including TB (36, 37).

**Figure 3:**
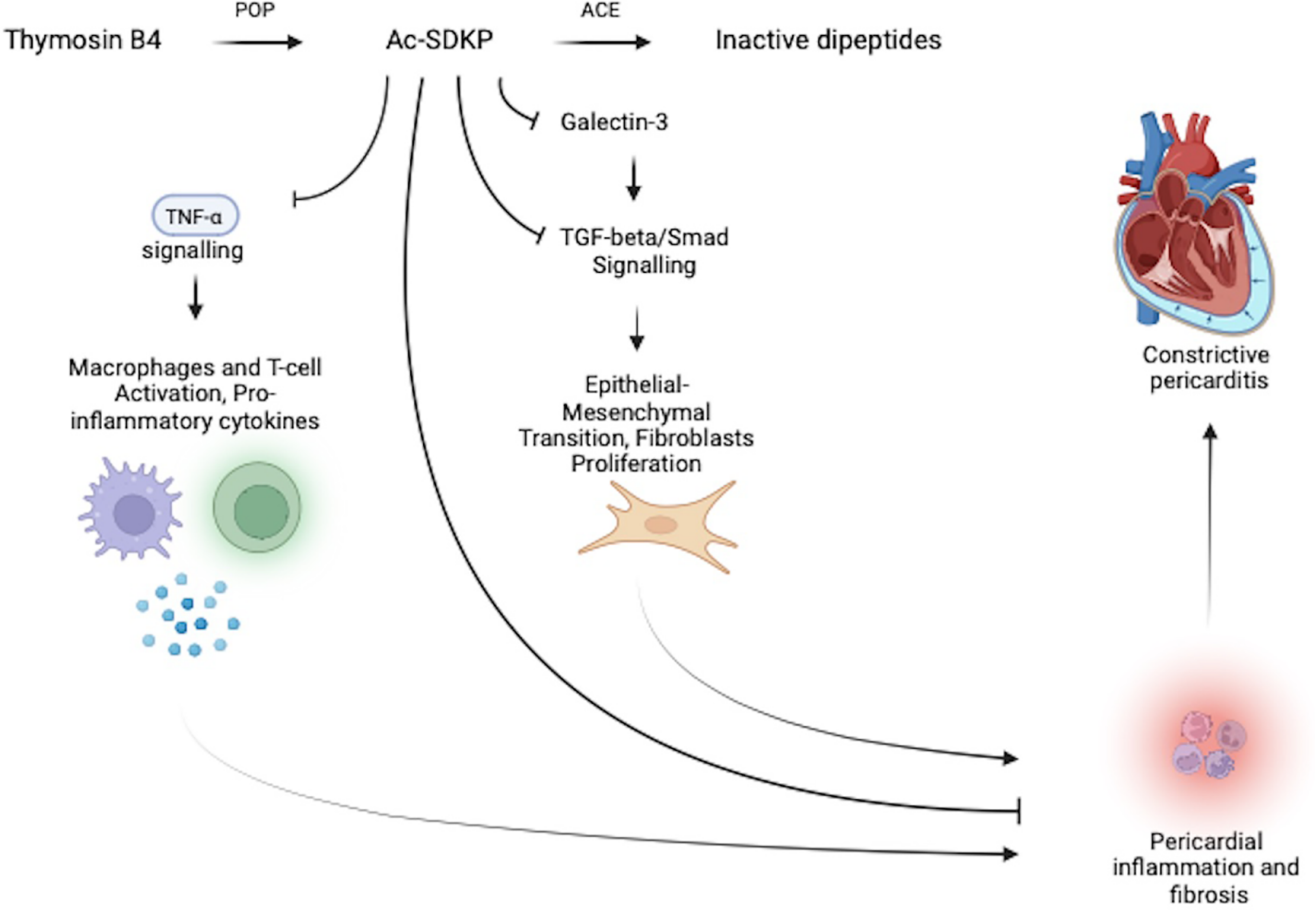
Pericardial housekeeping function of Ac-SDKP regulates pericardial inflammation and fibrosis. The anti-inflammatory peptide Ac-SDKP downregulates pericardial inflammation and fibrosis through its inhibitory effects on: (1) the galectin-3 induced TGF-β/Smad2 signalling pathway and (2) downstream and direct inhibitory effects on TGF-β, collagen synthesis, and TNF-α. Ac-SDKP is converted to its inactive peptide by ACE, which may allow for a convenient therapeutic target to upregulate both local (pericardial) and systemic Ac-SDKP levels through ACE-inhibitor therapy.

Ac-SDKP is a naturally occurring immunomodulatory peptide hydrolysed from thymosin β4 by prolyl-oligopeptidase (POP) and is converted to its inactive peptides by angiotensin-converting enzyme (ACE) (7, 35). It is hypothesised that Ac-SDKP serves a housekeeping function within the pericardium by inhibiting various major drivers of inflammation and fibrosis (see Figure 3). These include the inhibition of the galectin-3-induced TGF-β/Smad2 signalling pathway, a direct inhibitory effect on TGF-β, and a direct blocking effect on collagen synthesis. Ac-SDKP is also thought to inhibit TNF-α, resulting in a subsequent reduction of macrophage and T-cell activation and lower levels of pro-inflammatory cytokines (see Figure 3) (6–8, 35).

Furthermore, routinely available angiotensin-converting enzyme inhibitor (ACE-inhibitor) therapy was subsequently investigated and has proven effective in upregulating both local pericardial and systemic Ac-SDKP levels (7, 8), with the potential to play a pivotal role in promoting anti-fibrotic activity to reduce pericardial inflammation, fibrosis, and eventual pericardial constriction. This clinical application is still unexplored in current TB pericarditis literature.

It remains unclear whether this pro-fibrotic nature of the inflammatory response itself, the high intensity of the pericardial inflammation seen in TB pericarditis, the prolonged duration of the pericardial inflammation (due to the chronicity of TB), or another feature of the TB pathology *per se* causally drives the high incidence of pericardial constriction following TB pericarditis (38).

Although CMR does not specifically capture the pro-fibrotic nature of the inflammatory response, the intensity of pericardial inflammation can be accurately detected and quantified by evaluating the pericardial signal intensity on LGE imaging, making this CMR sequence an important modality capable of demonstrating this substitute marker of pericardial inflammation (39). Using a greater intensity of pericardial inflammation (in isolation) as a prognostic marker for developing pericardial constriction has however yielded conflicting results. While it has effectively demonstrated a more favourable prognosis by expressing the contribution of active pericardial inflammation in constrictive haemodynamics, often associated with the acute reversible phase of pericarditis (14, 15), it has also been associated with an increased risk of developing recurrent, chronic, and constrictive pericarditis (15, 16, 40–42).

Based on a study correlating CMR signals with histology, it has recently been suggested that a combination of pericardial inflammation and oedema signifies CMR evidence of acute pericarditis (16). In contrast, the absence of these two features defines a state of inert, burnt-out pericarditis (16). Between these extremes lie sub-acute and chronic pericarditis. Although both sub-acute and chronic pericarditis are associated with variable quantities of pericardial inflammation, sub-acute pericarditis is characterised by patchy areas of residual pericardial oedema, whereas chronic pericarditis is distinguished by the absence of residual pericardial oedema (16). Therefore, CMR can be used not only to quantify pericardial inflammation but also to stage the chronicity of disease by combining an analysis of inflammation (LGE) with that of oedema (T2-STIR and T2 mapping) (16). T2-STIR sequences, as well as T2-weighted mapping, are highly specific for detecting increased water content in tissues and can therefore identify pericardial oedema, a marker of acuteness and reversibility of pericarditis and potentially its complications, particularly when seen in the setting of residual pericardial inflammation (16).

In contrast with idiopathic pericarditis (39), TB pericarditis may display a prominent chronic pericarditis phase (absent pericardial oedema), combined with high-intensity residual pericardial inflammation, as illustrated in the following case.

## Case 2

A 26-year-old HIV-uninfected man presented with constitutional symptoms, exertional dyspnoea, and a clinical examination suggestive of a pericardial effusion. TTE confirmed a large, circumferential pericardial effusion conducive to pericardiocentesis, which was subsequently performed and confirmed a diagnosis of rifampicin-sensitive, definite TB pericarditis (17). This case was classified as ECP, as evidenced by the persistence of constrictive haemodynamics following successful pericardiocentesis (19).

**Figure 4:**
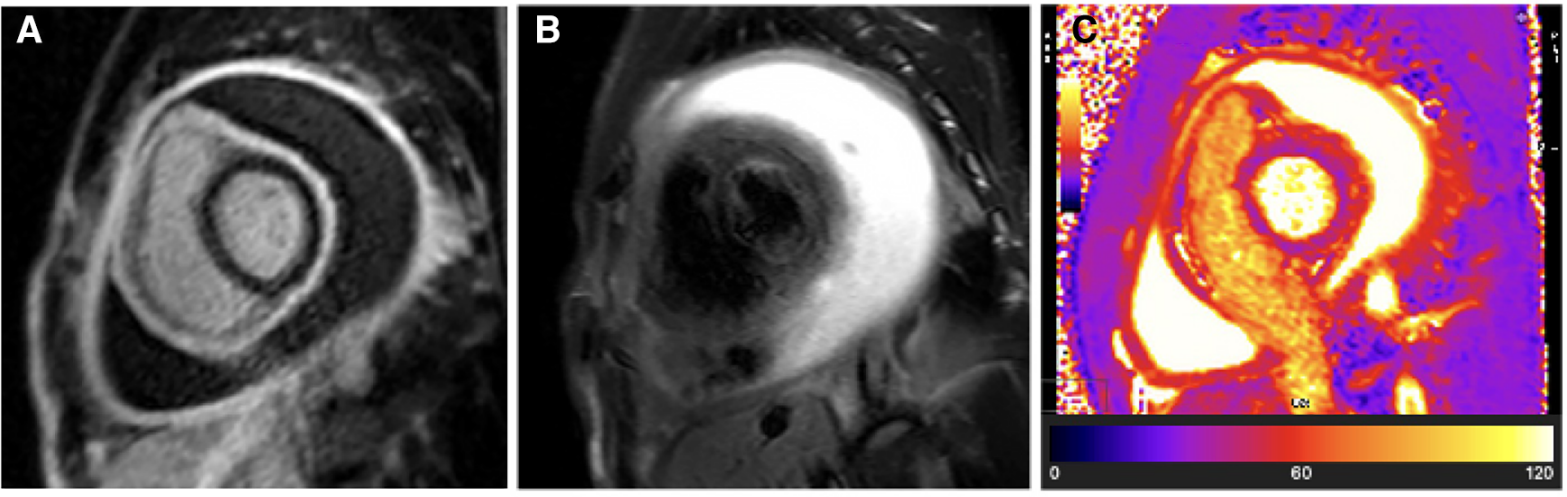
Case 2: Baseline CMR findings—chronic TB pericarditis with intense residual pericardial inflammation and a large recurrent pericardial effusion. (**A**) LGE image showing hyperintense circumferential pericardial signal indicative of intense circumferential pericardial inflammation. (**B**) T2-STIR and (**C**) T2 mapping images showing normal pericardial signal intensities indicative of absent pericardial oedema. The combination of (1) intense pericardial inflammation in the setting of (2) chronic pericarditis (absent pericardial oedema) likely represents the highest cumulative risk of developing constrictive pericarditis. Despite the visceral and parietal pericardium being separated by a large pericardial effusion, the pro-fibrotic “sticky” visceral and parietal pericardium is in the process of tissue remodelling and has a high risk of becoming adherent as the residual pericardial effusion resorbs. Compressive effects from the residual pericardial effusion still contribute to reversible constrictive haemodynamics; therefore, medical therapy needs to be continued until the pericardial fluid has completely resorbed.

A CMR was performed 6 days after the initial pericardiocentesis (baseline study), which revealed a large re-accumulated circumferential pericardial effusion. The CMR further illustrated intense residual pericardial inflammation on LGE sequences (see Figure 4A) but no associated acute pericardial oedema on T2-weighted STIR or T2 mapping sequences (see Figures 4B,C).

**Figure 5:**
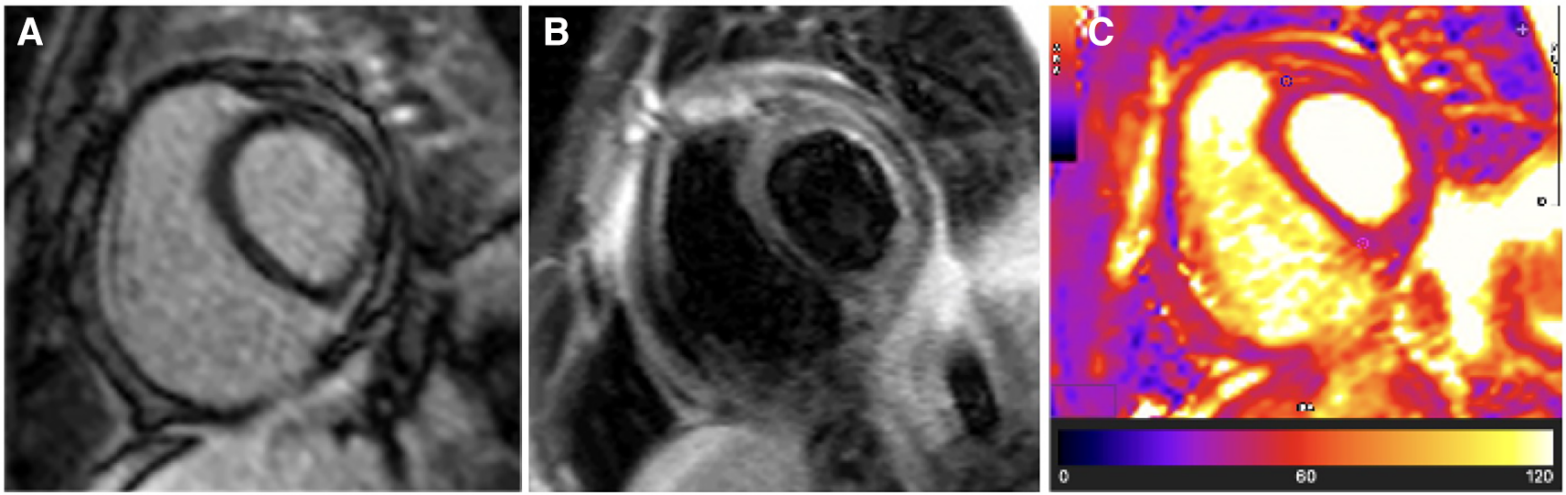
Case 2: 9-month follow-up CMR findings—burnt-out TB pericarditis. (**A**) LGE image showing the absence of a pericardial signal, demonstrating complete resolution of pericardial inflammation. (**B**) T2-STIR and (**C**) T2 mapping images showing normal pericardial signal intensities, demonstrating the absence of pericardial oedema. A burnt-out (resolved pericardial inflammation and oedema) CMR picture of TB pericarditis in the setting of constrictive haemodynamics is an indication for pericardiectomy.

The patient was initiated on the local first-line anti-tuberculous chemotherapy regime (20) without adjunctive anti-inflammatory medication and was followed up at a primary healthcare facility. Around the time of completing his anti-tuberculous chemotherapy (6 months after treatment initiation), the patient was noted to have developed progressive signs of predominantly right-sided cardiac failure. Due to poor access to transport and a congested medical service in a resource-limited setting, specialist follow-up was delayed, and the patient was only seen at a cardiology service 3 months later. At this stage, the TTE confirmed constrictive haemodynamics, and a repeat CMR study demonstrated complete resolution of the prior intense pericardial inflammation observed on LGE imaging (see Figure 5A). No associated pericardial oedema was present on either T2-STIR or T2 mapping sequences (see Figures 5B,C), suggesting that a burnt-out pericardial constriction phase was entered with little to no expectation of spontaneous resolution of the pericardial constriction.

The patient was subsequently referred for early pericardiectomy, which confirmed constrictive pericarditis with organised fibrosis of the pericardium demonstrated on histology.

### Case 2: discussion

Employing CMR to stage and quantify pericardial inflammation could potentially assist not only in prognosticating patients at higher risk of tissue remodelling and pericardial constriction but also in predicting the degree of disease reversibility, tailoring medical therapy, and determining the ideal timing for surgical pericardiectomy (9–16).

**Figure 6:**
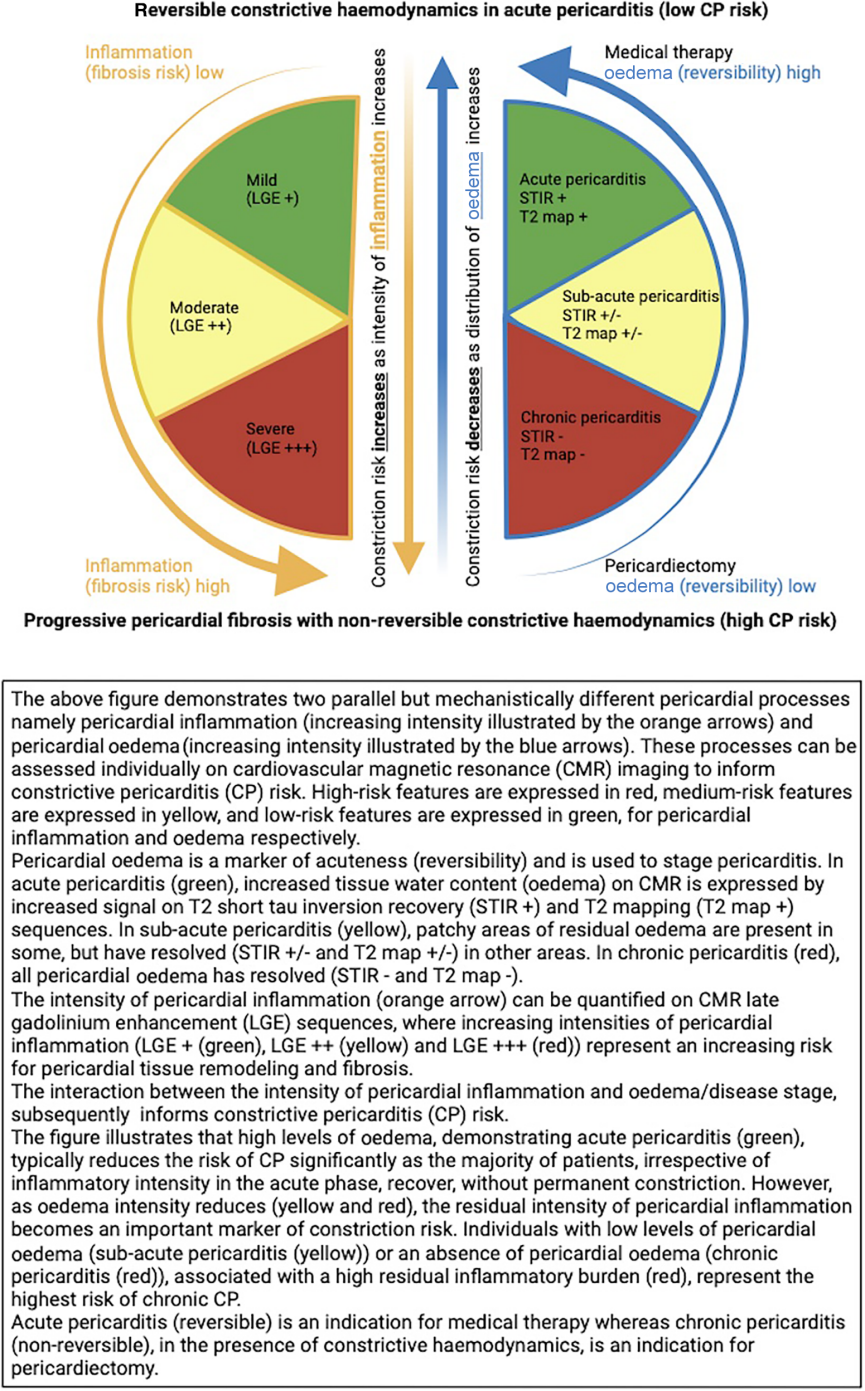
CMR-based model to predict constrictive pericarditis risk—a product of pericardial inflammation and oedema.

When choosing the most appropriate therapy, the stage of pericardial inflammation (as indicated by the presence of residual pericardial oedema on T2-STIR and T2 mapping) could be used to predict the reversibility of constrictive haemodynamics, while the intensity of pericardial inflammation (as quantified on LGE sequences) could be used to predict risk for continued pericardial tissue remodelling and fibrosis (see Figure 6) (14–16).

Staging pericarditis offers a potentially elegant solution to the previously mentioned discrepant findings seen in the CMR-derived pericardial inflammatory signal to predict constriction risk. Based on this staging model, oedema (T2-STIR and T2 signal) tracks acuteness, whereas inflammation (LGE signal), although typically present in the acute phase, can also extend into the sub-acute and chronic phases (16). Therefore, an analysis of the stage of pericarditis may be required in conjunction with the inflammatory intensity to contextualise the inflammation and subsequent constriction risk (see Figure 6).

The current best evidence suggests that, in acute pericarditis, an acute inflammatory response combined with pericardial oedema causes the pericardium to stiffen and become less compliant (16). Depending on the intensity and distribution of inflammation and oedema, this initial inflammatory response can result in decreased pericardial compliance and transient constrictive haemodynamics (16). However, even though the acute stage is transient and typically reversible, the intensity of inflammation seen in this stage may predict the future risk of dysfunctional, fibrotic healing and persistent constrictive haemodynamics, i.e., chronic CP (15).

In sub-acute pericarditis, oedema is seen to have resolved in some, but not all, pericardial segments, leaving patchy areas of the T2 signal where oedema persists and actively contribute to constrictive haemodynamics (16). Areas with residual inflammation but resolved oedema are thought to represent a stage in the process of healing, which can occur either with or without the formation of visceral–parietal adhesion—once again, the risk of healing with fibrosis is likely dependent on the intensity of the initial inflammatory response during the acute stage of the disease (15, 16).

Finally, in chronic pericarditis, inflammation of varying intensity may persist, along with the potential for ongoing tissue remodelling and fibrosis. Oedema is observed to have completely resolved; therefore, areas with established pericardial fibrosis are permanent and irreversible (16). In this chronic stage of the disease, if constrictive haemodynamics is present, it is likely to be irreversible, and pericardiectomy is indicated.

## Constrictive haemodynamics—an added prognostic opportunity?

The permanence of constrictive haemodynamics needs to be assessed within the context of its associated pericardial inflammation (on LGE imaging) and oedema (T2-STIR or T2 mapping), i.e., stage of the pericarditis (16). While constrictive haemodynamics might be transient and reversible in acute pericarditis, the presence of constrictive haemodynamics in sub-acute and chronic pericarditis represents two points on the spectrum of ECP, where the relative contribution from acute (and subsequently reversible) constrictive haemodynamics becomes sequentially smaller, while the contribution from established pericardial scarring (and subsequently non-reversible constrictive haemodynamics) becomes progressively larger (16). Therefore, the presence of constrictive physiology at any stage of the disease, particularly in later stages, is likely a sign of increased pericardial fibrosis burden and represents a state of impaired physiological diastolic reserve brought about by established pericardial scarring, even in the absence of a final constrictive outcome. However, the sooner a patient transitions from constrictive to non-constrictive haemodynamics, i.e., transitions in the acute rather than the sub-acute stage, the less significant the limitation of their final physiological diastolic reserve is likely to be.

## T2-weighted STIR—an indicator of reversibility or merely a marker of chronicity?

The reversibility of constrictive haemodynamics, as assessed by both (1) the intensity of pericardial inflammation (LGE imaging) and (2) pericardial oedema (on T2-STIR and T2 mapping), remains poorly explored. Prior studies that evaluated the contribution of active pericardial inflammation in constrictive physiology were conducted in the “pre-staging” era of pericarditis (14, 15). Notably, these studies only evaluated the contribution of LGE, a significant methodological limitation that may now be overcome in the era of multi-parametric CMR that includes oedema imaging and T2 mapping. Although one would expect a relatively linear relationship between an intensely inflamed pericardium and pericardial oedema and that oedema would resolve as the intensity of inflammation subsides, a clear disconnect between inflammatory and oedema signals is commonly observed in the context of TB pericarditis. This then begs the question of whether residual pericardial inflammation, in the absence of associated oedema (chronic pericarditis), represents residual reversible haemodynamics. Further research utilising multi-modal CMR in regions with a high burden of TB is clearly required.

## Conclusion

Although there remains uncertainty regarding the relative contribution of isolated pericardial inflammation (the absence of oedema) to reversible constrictive haemodynamics, there is no doubt that CMR may add significant diagnostic value in complex clinical cases of pericarditis. This becomes especially relevant in low- to middle-income settings like sub-Saharan Africa, where TB pericarditis is prevalent and carries a high inherent risk of progression to pericardial constriction. Further studies are required to explore the relative contributions of pericardial inflammation and oedema as mutually non-exclusive entities (may co-exist without clear linearity), contributing to the final constrictive risk. The apparent disconnect between marked residual pericardial inflammation in the setting of chronic TB pericarditis (in the absence of pericardial oedema) needs to be further researched to determine its role as a potential catalyst underpinning the disproportionately high risk of constrictive pericarditis observed in individuals with TB pericarditis.
